# Human limb position sense measured by repositioning during changes of gravity in parabolic flight

**DOI:** 10.1038/s41598-025-23941-9

**Published:** 2025-10-15

**Authors:** Bernhard M. Weber, Michael Panzirsch, Harsimran Singh, Uwe Proske

**Affiliations:** 1 German Aerospace Center, Institute of Robotics and Mechatronics, 82234 Wessling, Germany; 2 School of Biomedical Sciences, PO Box 13F, Monash University, Clayton, 3800 VIC Australia

**Keywords:** Position sense, Proprioception, Microgravity, Hypergravity, Parabolic flight, Neuroscience, Physiology

## Abstract

Our sense of limb position is believed to be signalled by muscle spindles. To confirm this, we previously sought evidence for spindles contributing to each of three different methods of measuring position sense. These included two-arm matching, one arm pointing and repositioning. The method of repositioning showed little evidence of spindle involvement. This study aims to seek further supporting information for the absence of a spindle contribution to repositioning. With this method, the passive limb is moved to a chosen joint angle and the blindfolded participant is asked to remember the angle and, subsequently, to reproduce it. It is known that responses of spindles are disturbed by changes in gravity. The present experiments were carried out during rises in gravity (hypergravity) and during falls (microgravity), in parabolic flight, to determine whether this disturbed position sense by repositioning. In order to maximise effects, the remembering stage, was carried out in one gravity state and repositioning in a different state. It was found that repositioning accuracy was unaffected by changes in gravity. These results suggest that the method of repositioning, used for measuring position sense, relies on a central memory of previous positions, rather than on peripheral input from muscle spindles.

## Introduction

Our position sense is the ability to sense the position of different parts of the body relative to one another and to their environment. It is one of the proprioceptive sensations, which include the senses of movement, effort, force and balance^1^.

### Spindles as position sensors

It is now well accepted that muscle spindles provide us with our position sense, with a supporting role from skin and joint receptors. For a comprehensive discussion of this issue, see^1^. Spindles are stretch receptors. Mammalian spindles typically comprise two types of sensory ending contained within a single capsule, the primary ending and the secondary ending. The primary ending is thought to signal both changes in muscle length and the rate of length change. Secondary endings predominantly signal changes in length. The rate sensitive component of the primary ending’s response is believed to underlie movement sense, while both primary and secondary endings are thought to contribute to position sense, based on their ability to maintain steady levels of discharge at different muscle lengths. It is believed that although position sense and movement sense both have their origin in signals from muscle spindles, they are distinct sensory entities which are processed independently by the central nervous system^2^. Discharge variability measurements for the two sensory ending types, measured in the presence of fusimotor activity, have led to the conclusion that over a muscle’s full-length range, primary endings would be able to signal only 6 different lengths. The figure for secondary endings was 25 lengths, indicating the large difference in discharge variability between the two ending types^3^. Such evidence supports the idea that the principal position sensors are likely to be the secondary endings.

Since a number of different methods are used to measure position sense, in a previous study we asked the question, do spindles play a role with all of these methods^4^? We have chosen three methods, aspects of which account for most of the reported measurements^4^. Unexpectedly, we encountered a method which, at least at first sight, did not appear to depend on ongoing levels of spindle discharges in muscles of the limb whose position was being measured. Because this finding goes contrary to the conventional view, we decided to study it in more detail.

The three methods chosen for measurements of position sense included that first used to reveal a contribution of spindles to kinaesthesia, the method of two-arm matching^5^. Here the blindfolded participant has one forearm placed at a chosen test angle and they are asked to align their other arm in a matching position. During vibration of elbow muscles of the reference arm, illusions of movement and displacement were perceived by the participant, as signalled by their positioning of the non-vibrated arm. Since spindles are vibration sensitive, it was concluded that they were able to generate kinaesthetic illusions, including illusions of displaced position.

The second method chosen was that of one-arm pointing. This is similar to two-arm matching, but here position sense is measured only in one arm, which is hidden from view. The participant holds a pointer in their other hand with which they point to the perceived position of the hidden arm. This is, therefore, a measurement of proprioception in only one arm, not a comparison between signals from both arms. For both the method of two-arm matching and one-arm pointing there was unambiguous evidence of spindles participating in the generation of the position signal.

The third method studied was that of repositioning, a method frequently used by clinicians because of its accuracy and ease of application. Here the blindfolded participant has their forearm placed at a chosen test angle and they are asked to remember this position. The arm is then returned to its starting position and, after a few seconds, the participant is asked to reposition the arm at the remembered angle. This method therefore involves memory and, in that sense, is distinct from the other two. Interestingly, the evidence for participation of spindles in the repositioning process was weak^4^ and we were unsure whether they made any contribution at all. It therefore deserved further scrutiny.

### Testing for a spindle contribution using thixotropy

When it emerged, that spindles appeared to play little or no role in repositioning, an experiment was designed to try to specifically bring out any aspects that could be traced back to spindles^4^. Here the method of thixotropic conditioning was used^6^. It provides the facility of generating different levels of spindle discharge at the same muscle length. Spindles are unique amongst sensory receptors in possessing this property, a consequence of their afferent terminals lying on striated muscle. All striated muscles, including intrafusal fibres, exhibit thixotropy. Therefore, by applying thixotropic conditioning to the method of repositioning, we aimed to reveal a contribution, if any, from spindles to generation of the position signal.

Thixotropy in a resting muscle is a consequence of the presence of a small number of stable cross-bridges between actin and myosin filaments in sarcomeres^6,7^. These stable bridges begin to form immediately after a contraction. When a muscle is held at a long length, stable bridges will have formed at that length. If the muscle is then shortened, the stable bridges do not immediately detach and they act as a splint on muscle fibres which are therefore unable to shorten incrementally. It leads muscle fibres to fall slack. The resulting low passive tension in slack intrafusal fibres reduces the maintained strain on spindle sensory endings and lowers spindle responsiveness to length changes. Therefore, depending on whether measurements are made after a contraction or after a stretch, the accompanying spindle sensitivity, made at the same length, will be different. This provides the opportunity for manipulating spindle sensitivity in experiments.

The way we have tested for thixotropic behaviour was to make measurements of position sense after a voluntary contraction or after a shortening movement from a longer length. Spindle rates will be high (after a contraction) or low (after a shortening movement) and any position errors which are correlated with these changes are considered to have spindle signals involved in their generation.

The method of repositioning is a two-step process. First, the experimenter presents the blindfolded participant with a chosen test angle at the forearm and asks them to remember it. Then, after they have returned their arm to its starting position, following a short delay, they are required to replace the arm at the remembered position. The method we used to try to reveal any spindle influences in this process^4^ was to present the test angle, for the memorising stage, with elbow muscles and their spindles in one thixotropic state. After participants had returned the arm to its starting position, the thixotropic state was altered, so now in their search for the correct angle for repositioning, participants were confronted by arm muscles with altered spindle discharge rates. That is, if spindle signals coming from elbow muscles, were involved in the repositioning process, changing spindle rates between the memorising and repositioning stages was expected to increase the size of any position errors. In the event, in this experiment position errors in repositioning continued to be small and there was no evidence that the thixotropic manipulation had had any effects^4^.

### Position sense disturbances during parabolic flight

It has been known for some time that astronauts experience a disturbance of their position sense under conditions of low gravity. The current view is that this is a consequence of gravity-dependent changes in spindle responsiveness interfering with position sense^8,9^. When the opportunity arose to make measurements during parabolic flight^10^, all three methods of position sense measurement were tested. While the likely participation of spindles in two-arm matching and one-arm pointing was confirmed, based on significant effects of hyper and microgravity on position errors, there was no effect of gravity on position sense measured by repositioning.

As a confirmatory test for this finding with repositioning, we have used gravity changes in a similar way to the thixotropic tests. During a parabola, at a given muscle length, there are anticipated changes in spindle rates, both decreases (microgravity) and increases (hypergravity). Any position error distributions which correlated with the anticipated gravity changes were postulated to be under the influence of spindles.

We measured repositioning errors in normal gravity (horizontal flight, NG), hypergravity (ascending and descending phases of a parabola, HG) and in microgravity (flight across the peak of the parabola, MG). Here we assumed that if there were any gravity effects on repositioning, the values measured during NG would be significantly different from those during HG or MG. That was not the case, there were no significant differences between the three measurements^10^. But before concluding that repositioning was impervious to gravity changes, we wanted to try to carry out an experiment resembling the ground level experiment with thixotropy^4^.

In the first parabolic experiments^10^ we had exposed both phases of repositioning (memorizing and reproducing) to the one gravity state. A stricter test would have been to carry out the memorizing phase in one gravity state and then the reproduction phase in a different state. By exposing the two stages of repositioning to different gravity phases, if there was any spindle contribution to repositioning, we expected to see larger position errors. The present experiments were designed to specifically test this point. Our working hypothesis was that for measurements of position sense by repositioning, exposing the two phases of measurement to different gravity states would not reveal new, previously undetected errors.

A rather broader objective of the present study was to try to obtain a better understanding of the disturbance of position sense reported by astronauts during conditions of weightlessness^11^. If we are right, and repositioning does not directly involve spindles in the generation of position sense when this method is used, it represents an important, new insight. It means repositioning tasks are able to be carried out, unimpeded during changes in gravity. On the other hand, the likely contribution of spindles to position sense by matching or pointing must imply it is during these kinds of tasks that disturbances will be revealed. If so, this points to a possible resolution of the problem. Normal spindle discharge rates may be recoverable in microgravity by, for example, the wearing of elastic garments that re-establish normal torque levels in muscles and tendons. These matters are discussed in the present study.

## Methods

In these studies, measurements were all carried out at the forearm, in the sagittal plane, in the absence of vision. They were therefore strictly proprioceptive tests.

### Sample

**Fig. 1:**
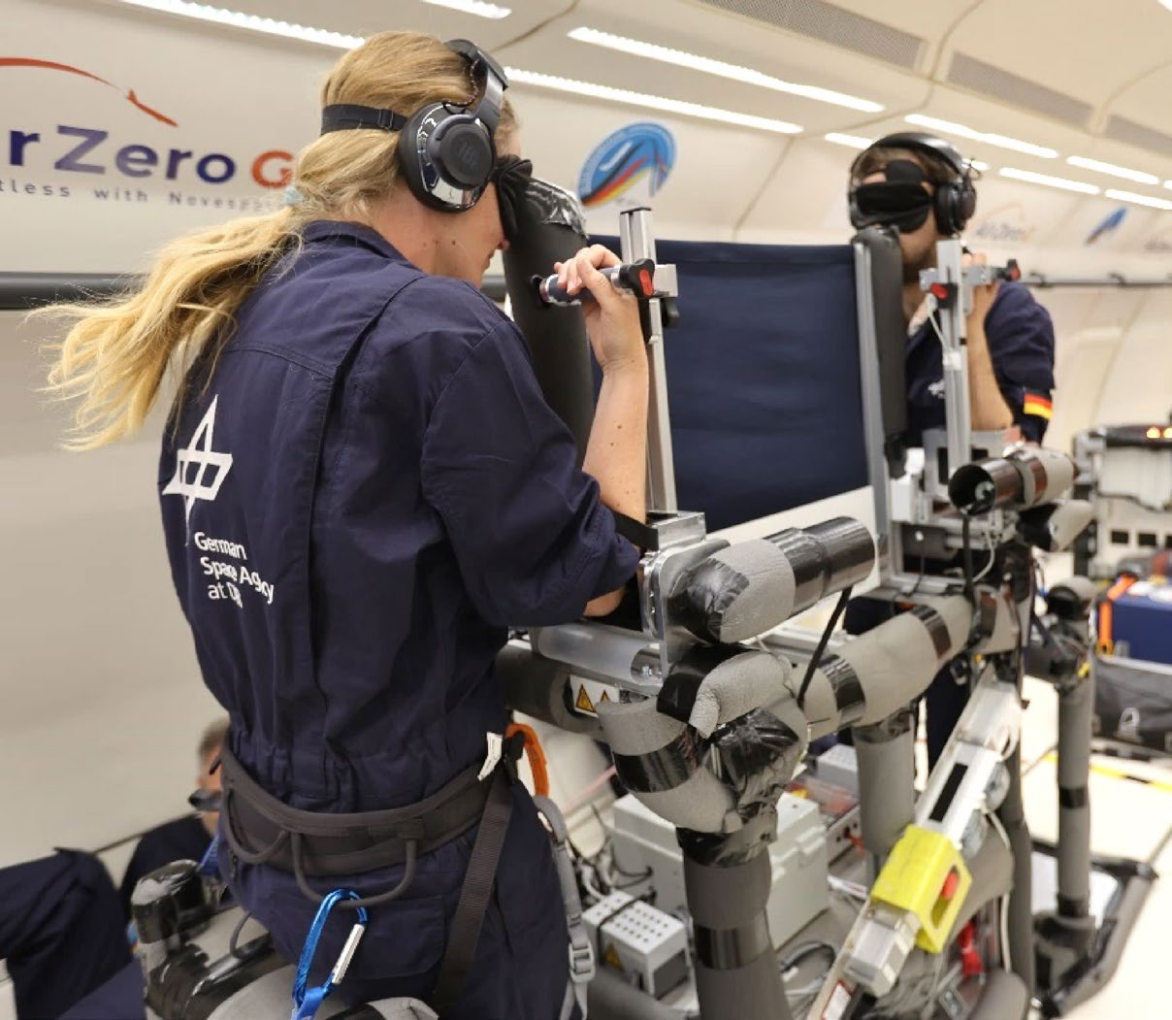
The experimental setup installed in the airplane. Two participants performed the experimental task simultaneously. Both participants gave written consent for publication.

Six participants (1female, 5male; 5 right-handed, 1 ambidextrous) 30–47 years old (M = 40.3 years, ± 5.7 SD) took part in the study. Participation was voluntary and unpaid. All participants had taken part in a similar parabolic flight campaign one year before, so they were familiar with what was required. All participants had fulfilled the medical requirements for participating in a parabolic flight campaign. No participants reported carrying out regular fitness exercises with their arms, nor were they carrying any recent arm injuries. Their health status was re-checked one day before their flight by an aviation physician. Participants were thoroughly informed about parabolic flight, safety and emergency procedures, medication, experimental tasks and procedures. All participants provided written, informed consent. In addition, the two individuals depicted in Fig. 1 gave their informed consent for the image to be used in an open access publication. The experimental protocol was approved by a French ethics committee (Institute Paoli Calmettes, “Comité de protection des personnes Sud-Méditerranée I”; ID RCB number: 2023-A01115-40) and all ethical aspects conformed with the Declaration of Helsinki.

### Apparatus

#### Experimental setup

The experimental setup comprised three structural components: one main rack with two pairs of paddle setups installed on the top of it and two seats, each mounted on the front of the main racks (see^12^, for a technical description of the setup). All the three components were designed to be mechanically robust even in the event of an emergency landing (acceleration of ~ 9 g) and were firmly bolted to rails on the aircraft floor. The two paddle setups were installed on the main rack, facing each other, so that two participants could perform the experiment simultaneously (see Fig. 1). The paddle setup and the seat heights could be adjusted individually to suit the participants. Each participant was standing in front of the two paddles for both arms, which could be moved in the sagittal plane. The movement range of each paddle was 120° from the horizontal (0°) to a flexed position (120°), with mechanical stops preventing movement beyond these limits.

Motors were mechanically coupled to the paddles to automatically move them to pre-defined angles. One of the two paddles on each side was additionally equipped with an electromagnetic clutch integrated between paddle and motor. Opening this clutch allowed free paddle motion since the motor was now fully decoupled. Free motion of the paddles without any accompanying frictional or inertial effects was important for indicating angle positions. Potentiometers (Contelec, WAL305 5 K, angular resolution of ± 0.3°) attached to each paddle’s axis of rotation, recorded the indicated angles with a sampling frequency of 1 kHz. The paddle motors were also equipped with motor brakes, allowing them to be locked in any paddle position. The participants’ arms were placed on the paddle with elbows resting on a soft support (a Velcro strap held the elbow in position) while the hand held a handle at the end of the paddle. The distance between elbow rest and handle and between handle and paddle could be individually adjusted.

The participants’ feet were strapped to the base plate of the main rack for stabilisation in microgravity. The seat support behind the participants stabilised them during hypergravity episodes. The experimenters monitored the experiment from the side of the main rack. Their workplace was equipped with a laptop for monitoring and controlling the experimental procedures.

#### Experimental software

The setup was equipped with an inertial measurement unit (IMU) to detect the different gravitational episodes during a parabola (1.8 g, 0 g, 1.8 g, 1 g). An experimental trial was started in the initial hypergravity phase (“HG1”) of a parabola after gravity values exceeded 1.5 g; the microgravity phase (“MG”) was detected after they fell below 0.6 g, and the second hypergravity phase (“HG2”) when the g-value was back above 1.3 g. The normal gravity (NG) phase at the end of the parabola was then triggered when g-values had returned to below 1.15 g (Fig. 3). For each of the four gravity changes, a time limit of 5 s had to be exceeded before the next experimental step was initiated. When this happened, participants received the corresponding experimental instructions via headphones. The timing of the pre-recorded audio instructions was controlled by a JAVA program. The software for IMU data analysis and the control of the paddles was implemented in Matlab/Simulink. It was executed on a real-time Linux system at a sampling rate of 1 kHz.

#### The experiment

**Fig. 2:**
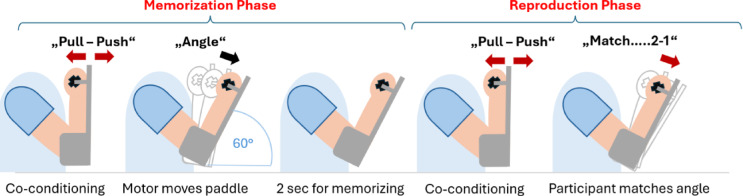
The repositioning procedure. There was a memorization and a reproduction phase, each started with co-conditioning of elbow flexors and extensors.

At the beginning of the experiment, participants were blindfolded by the experimenter. All further steps in the one-arm repositioning task were carried out with the arm strapped to the paddle which had the integrated clutch. The initial position of this paddle was 90° (vertical). The other arm was attached to the other paddle and was not moved throughout the experiment. The repositioning always consisted of the following two steps (see Fig. 2): (1) Memorization: The participant co-conditioned both antagonists of their arm (elbow flexors first, extensors second) with isometric contractions. The contractions were approximately 20% of maximum, 0.5s in duration. Once they had relaxed, the paddle was automatically moved by the integrated motors from the vertical starting position to the test angle position. The arm was held at that angle for 2 s while the participant remembered its position. (2) Reproduction: The paddle automatically moved back to the vertical starting position and the arm was again co-conditioned. Then, the clutch of the paddle was released and the participant was asked to move the arm to the remembered position.

**Fig. 3:**
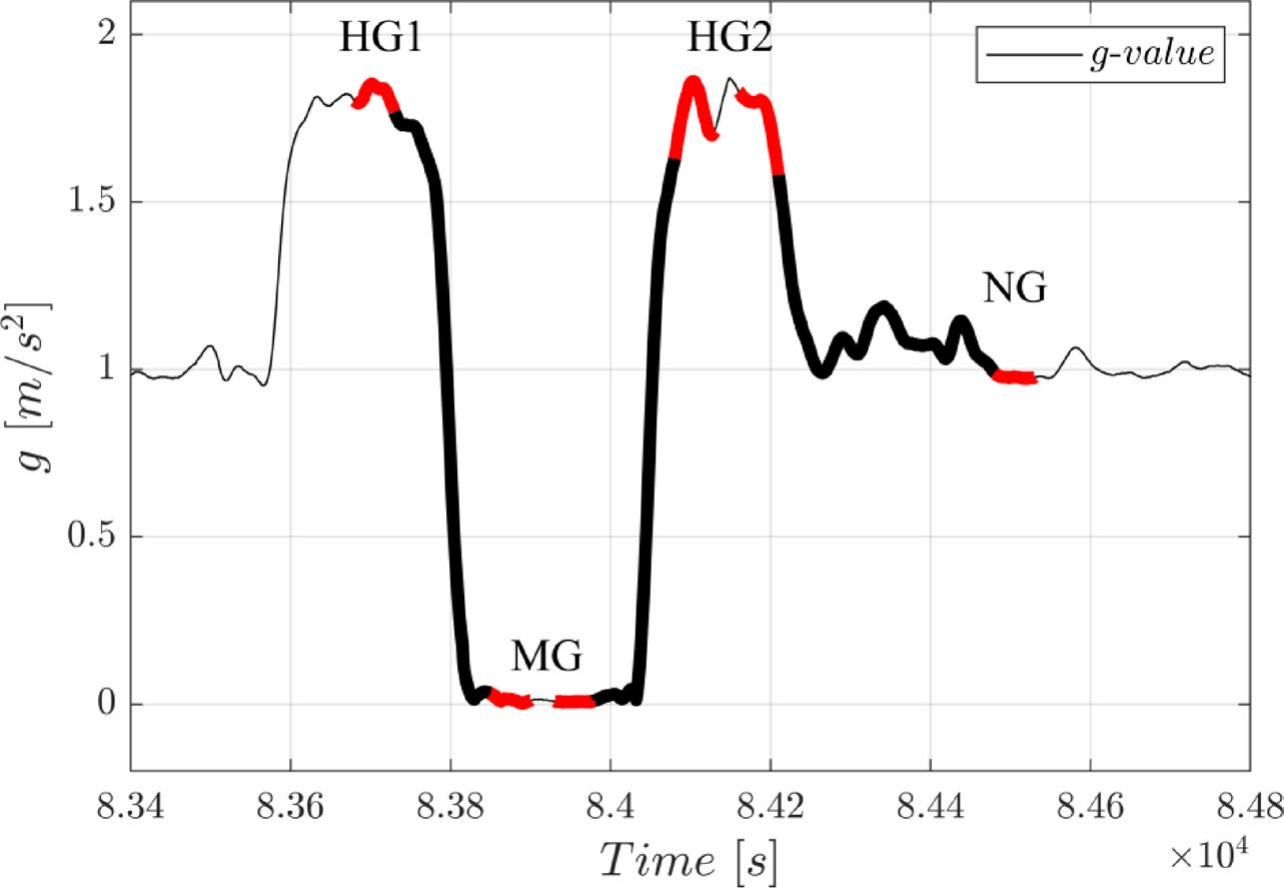
Example of g-value recording for one parabola (#26). The thick lines indicate time periods for the three repositioning trials per parabola (HG 1 → MG, MG → HG2, HG2 → NG). The respective memorization and reproduction phases are shown in red.

For this experiment the test angle used was always 60°, representing approximately the middle of the forearm’s working range. The repositioning procedure was performed three times, while the memorization and reproduction steps were carried out during different gravitational phases (see Fig. 3). In the initial trial, memorization was performed during the first hypergravity phase (HG1) and reproduction in the following microgravity phase (MG). In the same MG phase, the second trial started with the memorization step and it was reproduced in the second hypergravity phase (HG2). Finally, the test angle was presented to the participant during the same hypergravity phase (HG2) and its position was reproduced when the subsequent normal gravity phase (NG) had been reached.

All instructions were pre-recorded. Co-contractions in the vertical position were announced with “pull-push” (see Fig. 2). Then, the test angle was presented with the announcement “angle”. As soon as the clutch on paddle opened, the instruction “match” followed. Participants had 5 sec to carry out the reproduction of the remembered angle. For the last two seconds there was a countdown “2 − 1”. Then the current angle of the paddle was recorded. This strict procedure was necessary given the temporal constraints during each parabola, with only 20–22 second duration episodes of hyper- and microgravity available.

#### Experimental design

A within-subject design was implemented and each participant completed two experimental sessions: a pre-flight session one day before the flight and a flight session. During the flight session, the experiment was carried out over ten parabolae. During the pre-flight session the same experimental protocol and procedure was implemented as during flight and results served as a comparison baseline for the flight results. However, the pauses of about 1 min 45 s between parabolae in flight, was shortened to 40 s in the pre-flight session. Also, after a block of five parabolae there was a pause of 5 min in the flight session. This pause was shortened to 60 s in the pre-flight session.

During each of the ten parabolae each participant completed three repositioning trials (1. HG1: memorization – MG: reproduction; 2. MG: memorization – HG2: reproduction: 3. HG2: memorization – NG: reproduction). This made for a total of 30 trials. The delays between memorization and reproduction phases of each trial were dependent on the parabolic flight maneuvers and on average the delays for the first two transitions were 17 s, while the corresponding delay for the last was 35 s, as it took a longer time to transition to stable, horizontal flight. The same applied to the pre-flight session, the timing of which was also based on g-level sequences of real flight data.

#### Procedure

One month before flight, all participants were informed in an online briefing about the background of the study, the experimental task and the parabolic flight. Over a period, 4–5 weeks before flight, participants were also invited to the German Aerospace Center in Oberpfaffenhofen for a detailed briefing about the experimental and safety procedures. Finally, participants completed an entire experimental session to serve as a rehearsal for the flight campaign. The flight campaign was organized and carried out by the company NOVESPACE at Mérignac International Airport in Bordeaux-Mérignac, France. On arrival in the NOVESPACE facilities, experimenters and participants were briefed about general procedures and safety regulations in the facilities and during flight.

On the afternoon of the day before the flight, the pre-flight session was carried out in the aircraft while on the ground. There were three flight days and two participants took part on each of the three days. All experimenters and participants received a Scopolamine injection (0.5–1.0.5.0 ml, depending on sex, weight and past flight experiences) in the morning of the flight day. Scopolamine is an anti-emetic drug used during parabolic flights which minimises the risk of motion sickness. The known side effects can be drowsiness, dry mouth and blurred vision. However, in our experiments no participant reported any side effects of the medication. After medication, participants boarded the Airbus A310. The two participants took up their positions at the setup; they were secured with a climbing harness which was attached to the seat structures, their feet were secured in the foot straps and arms were strapped into the paddle setup. They put on the headphones and the blindfold. The temporal sequence of a parabolic flight was ~ 20 s hypergravity, ~ 22 s microgravity, again ~ 20 s hypergravity, and back to normal gravity (see Fig. 3). As described above, the IMU detected the point when the required g-value had been reached, so that the experimental trials could begin. After a pause of ~ 1 min 45 s, the next parabola was commenced. While during each flight day a total number of 31 parabolae (parabola #0 is typically used for familiarisation) were flown, the present experiment was carried out during the final ten parabolae (#21-#30).

#### Data analysis

The error for one-arm repositioning was calculated as the difference between the remembered angle and the reproduced angle (both measured in degrees). This error value can be negative, indicating a position error in the direction of arm flexion, or positive, indicating an extension error where the paddle was moved beyond the 60° target angle. Extreme outliers for the error value (> 3 IQR) would be excluded from analysis. The recorded error measures for the three transitions (HG1-MG, MG-HG2, HG2-NG) were averaged across the ten parabolae for the following analyses. On flight day 2, however, a technical problem with one motor controller occurred. This problem was able to be solved during flight; but instead of 10 parabolae, data from only 5 parabolae (#26–30) are available from the two participants for that day.

The error measures were analysed with repeated measures ANOVA (rmANOVA) comparing the three transitions in the pre-flight and the flight session (2 (session: pre-flight vs. flight) x 3 (transitions: HG1-MG, MG-HG2, HG2-NG) rmANOVA). As effect size, partial $\:\eta\:$^2^ was calculated. Normality was checked using Shapiro-Wilk test and sphericity with Mauchly’s test. Greenhouse-Geisser corrections were made if non-sphericity was indicated by this test. All tests were conducted with a 5% alpha level. Alpha levels of post-hoc comparisons were Bonferroni corrected and Hedges’ g was calculated for these comparisons. For all comparisons two-tailed tests were chosen.

## Results

In a first step, normality of the data distribution was checked and no significant deviation was found, either for the pre-flight data or for the in-flight data (Shapiro-Wilk test; all *W*s > 0.84). Moreover, no extreme outlier values were identified. As normality was given, a rmANOVA was performed on the averaged position errors during the three transitions in both experimental sessions. This rmANOVA revealed no significant overall effects or interactions between sessions and transitions (Main effects: Session: *F* (1,5) = 4.0; *p =*.10; $\:\eta\:$^2^ = 0.45; Transition: *F* (2,10) = 3.09; *p =*.09; $\:\eta\:$^2^ = 0.38; Interaction effect: Session x Transition: *F* (2,10) = 0.54; *p =*.54; $\:\eta\:$^2^ = 0.12). A post-hoc statistical power analysis revealed sufficient power of the rmANOVA (1-*β* > 0.99 for the Transition main effect) despite the small sample size.

Although there were no significant main effects, post-hoc comparisons were carried out. Regarding potential Session effects, no significant differences between pre-flight and flight sessions were found for the three transitions. Yet, a moderate effect size was found for Transition 3 (Transition 1: *t* (5) = 0.43; Hegdes’ *g* = 0.12; Transition 2: *t* (5) = 0.43; *g* = 0.13; Transition 3: *t* (5) = 1.49; Hegdes’ *g* = 0.68).

Next, post-hoc tests comparing the three Transitions were performed. Indeed, results revealed a significant difference between Transition 1 (*M* = − 0.38°; *SD* = 3.02°) and Transition 3 (*M* = −2.78°; *SD* = 3.08°; *t*(5) = 4.04; *p* =.03), showing a tendency for increases in errors in the direction of flexion across the transitions. Yet, this difference was only significant for the flight, but not for the pre-flight session. For the flight session, the error values were *M* = − 0.58° (*SD* = 3.38°) for Transition 1 and *M* = −4.03° (*SD* = 1.61) for the Transition 3 (*t*(5) = 4.09; *p* =.03, *g* = 1.30), also see Table 1.

**Table 1:** Means and SD of errors [°] for the three (gravity) transitions during pre-flight and flight sessions.

	Transition 1 (HG 1 → MG)	Transition 2 (MG → HG2)	Transition 3 (HG2 → NG)
Pre-flight	− 0.18 (3.09)	−1.29 (2.50)	−1.52 (4.98)
Flight	− 0.58 (3.38)	**-**1.81 (5.30)	−4.03 (1.61)

**Fig. 4:**
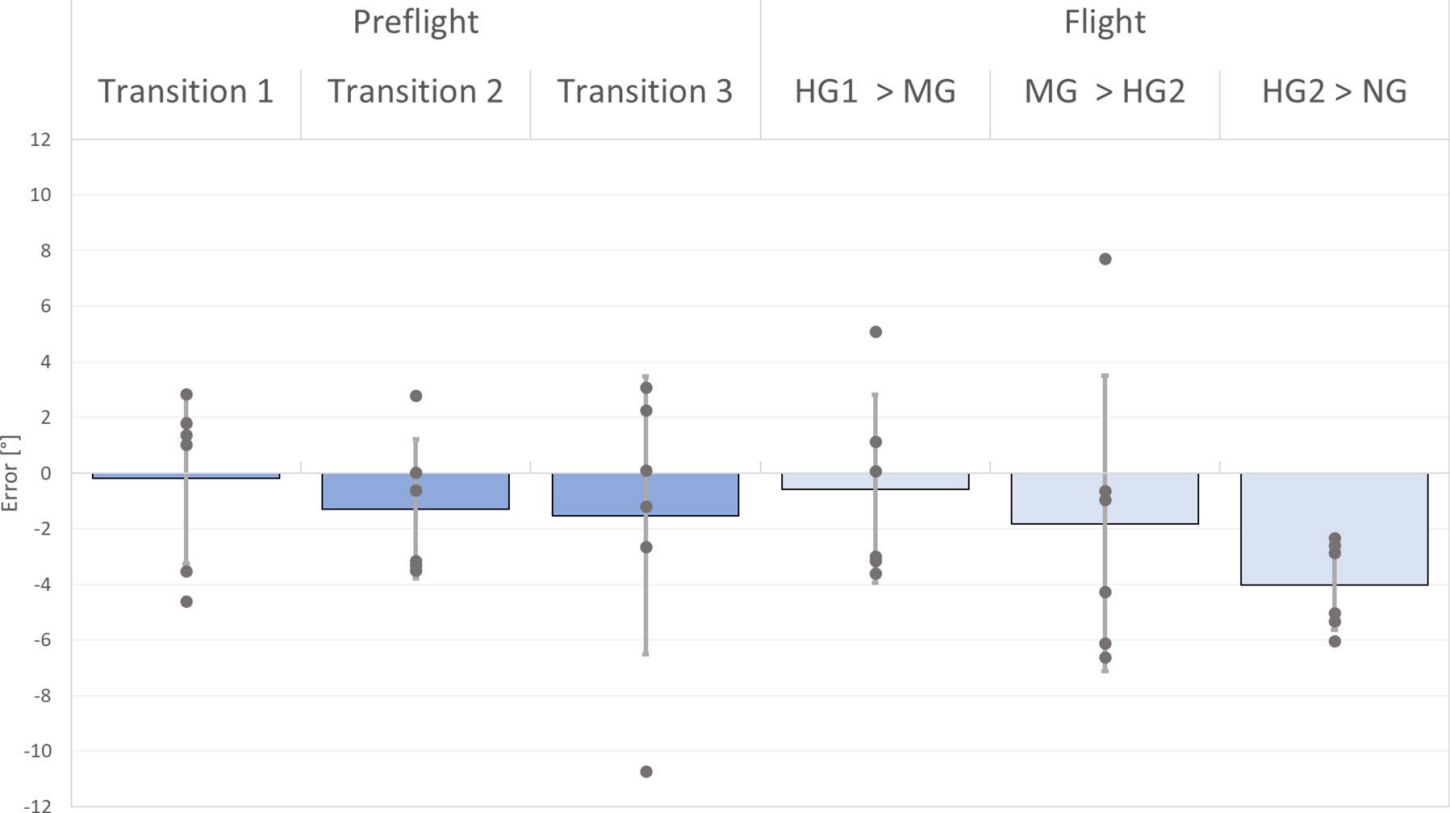
Repositioning errors during the different transitions for 6 participants. Display of the mean errors (± SD, shown by the error bars). Values for individual participants shown as grey dots.

In sum, the data revealed no significant difference between pre-flight and flight data. There were progressive increases in position errors, in the direction of flexion, during the three transitions, a trend that was particularly obvious during flight (Fig. 4). For this reason, the difference between the pre-flight and flight sessions was largest for the last Transition 3, with a moderate effect size.

## Discussion

The present study has shown that position sense accuracy, measured during a repositioning task, remains unaffected by changes in gravity generated during parabolic flight.

We have previously been studying position sense by repositioning and sought evidence for activity of muscle spindles contributing to position sense measured with this method. Our intention was to try to align repositioning with position sense measured by matching or pointing, where the evidence for the participation of spindles was unequivocal. To do that we used two methods of disturbing the afferents, thixotropic conditioning measured at ground level^4^, and gravity changes generated during parabolic flight^10^. The new experiments reported here were exclusively concerned with possible influences of changes in gravity on position sense during the repositioning process.

Following our first parabolic study^10^, we were not entirely satisfied with our position sense measurements for repositioning during parabolic flight and this led to the present experiments. During the earlier parabolic series, for repositioning, both memorizing and reproduction of the test angle had been carried out during the one gravity state. However, in order to better bring out possible peripheral influences, a more sensitive test would be to expose each repositioning stage to different gravity states. These measurements were made during three pairs of transitions in gravity, from HG1 to MG, MG to HG2, and HG2 to NG. During the first gravity state of each pair, participants memorized the test angle, while during the second state they reproduced it. Since we knew that changes in gravity evoked alterations in spindle discharges, this should have maximized any spindle effects on the repositioning measurements and therefore brought out evidence of their influence on the error distribution. In the event, no new, repositioning errors emerged. Our provisional conclusion, first put forward by Weber et al. (2025)^10^, that repositioning was insensitive to gravity changes, was supported by the results of the present experiments. We, therefore, reaffirm our view that neither changes produced by thixotropic conditioning, nor gravity changes altered error distributions measured by repositioning.

The ground-level experiments had provided only weak evidence for peripheral afferent activity contributing to the values of position sense measured during repositioning^4^. Yet other experiments had shown that, at a given muscle length, thixotropy could effect robust changes in steady discharge rates of spindles. For example, in animal experiments, for a single spindle, the change in steady discharge rate produced by thixotropic conditioning was 20 impulses s^−1^, which represented a three-fold change in rate between the two forms of conditioning, despite the fact that the muscle length at which these rates were measured remained the same (Wood et al.^13^, Fig. 1). Therefore, if thixotropy had an effect on repositioning, that should have been obvious from the distribution of the errors.

In our first experiments during parabolic flight^10^, there was evidence of significant alterations in position sense values measured by two-arm matching and one-arm pointing. For example, in a two-arm matching task, during an increase in gravity from 1G to 1.8G, position errors increased from a mean of 2.5° to 3.5°. During a fall in gravity from 1G to 0G, mean errors decreased from 2.5° to 0.35°. These changes in errors were significant and consistent with the hypothesis that the position sense mechanism for two-arm matching was gravity sensitive and that this was likely due to the role played by spindles in the generation of this sense. Similar trends were observed for measurements of position sense by pointing. By contrast, the values for position sense by repositioning were not significantly different during either normal, hyper or microgravity^10^.

Another difference that emerged from the ground-level experiments was that there appeared to be differences in accuracy between the three methods of measurement. For two-arm matching, the average error over the three mid-range angles at the elbow (35°, 65° and 95°) was 5.9° in the direction of extension. For one-arm pointing, it was 11.9° into extension and for repositioning it was 0.5° into extension^14^. It seems that a feature of the method of repositioning is that it is more accurate than both matching or pointing.

During parabolic flight, none of the values measured for repositioning in hyper or microgravity were significantly different from those measured during horizontal flight. This was regardless of whether the two steps in the repositioning process were both exposed to the same gravity state^10^, or to different states (present study). The conclusion that the repositioning mechanism does not involve access to ongoing spindle activity coming from muscles involved in the test appears, at first sight, to be heresy.

First, we considered the possibility that, as yet unrecognized, factors were responsible for the outcome. Considering the difficulties of making measurements in parabolic flight, some unexpected changes in errors were apparent. One example was the incremental increase in errors (non-significant) from Transition 1 to Transition 3. We are not certain about the sources of these changes. The time interval between the memorising and repositioning stages was rather long for the last transition, 35 s compared with 17 s for the other transitions. The longer delay may have influenced participants’ memory capacity, given the stressful in-flight conditions and unavoidable fluctuations in gravity values (Fig. 3). Moreover, potential order effects might play a role as the sequence of transitions was fixed. Other possible factors such as the low load on the arm and choice of the elbow joint as the test joint were unlikely to contribute to the outcome. For a discussion, see Proske (2024)^14^. In drawing our conclusions, we placed importance on the fact that two quite different experiments on repositioning, thixotropic conditioning and gravity changes, both led to the same outcome.

We don’t know why there is this difference between the three methods of measurement of position sense. Matching and pointing both show evidence of spindles participating in the generation of the position signal, but repositioning does not. Repositioning is the only method that makes use of a memory mechanism and it is this which must underlie the difference. Since memories are stored and retrieved from central sites, this process is not likely to involve direct inputs from peripheral sources. An important question is why is such a mechanism established, in the first place? Does the repositioning process subserve other, as yet unrecognized, roles? Furthermore, for the laying down of memories, some kind of initial calibration process involving peripheral afferents must take place. In the search for an explanation we have gone back into the literature on position sense^15,16^, seeking answers to our questions.

Horch et al.^15^ declared that it was generally assumed that static limb position sense depended on a continuous input from slowly adapting receptors whose activity levels were a function of joint angle. With the intention of obtaining more information about these receptors, a matching task at the knee was carried out, where one, passive, leg was placed at a given angle and the other had to match its position by active movement. It was found that participants could do this task quite accurately. However, during the experiments an alternative explanation for the generation of position sense occurred to the authors. It was proposed that participants were not using ongoing receptor activity coming from the passive reference leg. Rather, they remembered the position of the passive leg from its movement to the test angle, or soon after it had stopped. Direct tests of the capacity of participants to be able to remember a particular position of their leg, without continuously maintaining its position at the test angle, indicated that they could do this very well. Such a memory mechanism suggested an alternative explanation for static limb position sense; participants knew the position of their limb in the absence of movement because they had subconsciously remembered its position at the start of the movement and they used this to determine its current position. If this was so, there would be no need for continuous input from tonically discharging receptors in the leg to provide signals of static limb position^15^.

In a subsequent series of experiments carried out at the knee joint, Clark et al.^16^ were able to distinguish between position and movement senses by moving the leg at sufficiently slow speeds to be below the movement detection level. In order to detect a change in position of a joint, participants had to compare positions before and after a movement and judge whether a change in alignment of their legs had taken place. They did this without needing a reference to indicate the start of the movement. It meant participants must have depended on their memory of the starting position to determine the change in position. Since these movements were very slow, and trials could last up to half an hour, it suggested a relatively stable memory of joint position.

These observations suggest that whenever we make a movement, as soon as it stops, the position reached is immediately calculated, based on a comparison with the previous position, and this new value is automatically stored in memory. There is no contribution to this calculation from ongoing activity in proprioceptors and the new position is determined, purely, by what has gone before. The assumption is that the brain has access to different representations of the limb which may well be established, initially, by peripheral input, including that from muscle spindles^17^. However, once established, this representation can be addressed without referring to the current peripheral input from the muscles involved in the measurement. Such a proposition would account for measurement of position sense by repositioning being insensitive to any changes in ongoing afferent activity coming from the muscles involved in the test. As a consequence, repositioning errors remain immune to changes in afferent activity generated by either thixotropic conditioning or alterations in gravity. What impact might such a mechanism have on position sense measured by matching or pointing?

In a two-arm matching task, it is generally assumed that afferent signals coming from muscles of the reference arm are compared centrally (by a postural schema^18^ with signals from the indicator arm and the indicator moves into a position where the signal difference between the two arms is at its minimum^19^. This mechanism may need to be reassessed. The new proposal is that the participant knows the position of their reference arm by referring back to where it had come from and the known, adopted position is matched by the indicator arm. However, such an explanation does not account for the large, direction dependent, thixotropic errors seen in matching after appropriate conditioning of elbow muscles (± 6° of error, Roach et al.^4^, Fig. 2). Such errors, which are distributed approximately symmetrically, in the directions of flexion or extension, about the actual position of the limb, strongly suggest that the comparison is between afferent discharges coming from both limbs, not on memories of previous positions. To accommodate this, it is necessary to assume that the central nervous system adopts different strategies, depending on the choice of the method of measurement.

Our evidence suggests that both matching and pointing involve access to peripheral afferent signals during each test. It makes some sense to use spindle signals in two-arm matching. The signal coming from each arm tells the participant where the arms are and where the indicator arm must be moved to match the reference. Something similar is presumably happening in pointing. The location of the hidden arm is signaled by the pattern of spindle impulses coming from its two antagonists acting at the elbow joint. That pattern is converted centrally into a position signal, which is further converted into a visual signal to guide the pointing arm. When the task involves replicating an adopted position, as in repositioning, access to proprioceptive signals from the reference arm is not necessary. The moment the arm is placed at the test angle, its position will be remembered automatically. Therefore, we postulate that for the repositioning task it is not necessary to ask the participant to remember the test angle. They do that automatically, subconsciously, anyway. We plan to test this in future experiments. It remains to explain why such a very different mechanism is invoked when measuring position sense by repositioning, compared with matching and pointing. We suspect that there are as yet unrecognized roles for such a memory mechanism.

The continuous knowledge of the position of a body part, provided by a central memory process is likely to have broader implications than just giving convenient access to a mechanism for measuring position sense. An obvious question is how is such a mechanism generated? Can the process of referring back to previous movements be tracked right back to the beginning of the day? Clark et al.^16^ reported that for a test of position memory at the shoulder joint, participants revealed the ability to remember positions even after 24 h! That is, persistence of the memory appears to be long lasting. Another important question concerns the possible role of remembered limb position in motor control, such as reaching movements where the initial position of the limb, before the movement, plays an important role^20,21^. Is the position memory mechanism invoked here or are peripheral afferent mechanisms responsible? Finally, it would be interesting to study patients who are known to lack muscle spindles, such as in the conditions, HSAN III^22^ and Pompe disease^23^. We predict that such patients would have difficulty in carrying out matching or pointing tasks, that is, unless the role of spindles has been taken over by other receptors^24^. If such patients were able to carry out a repositioning task at all, then they should be able to do so without difficulty. But it is, of course, possible that neural mechanisms for the laying down of the necessary memories may be missing in such patients.

The repositioning mechanism may have a role in motor learning. When teaching a new motor skill, the instructor may often grasp the learner’s limb and guide it through the desired movement. In a study of the relationship between the learning effect of passive training and proprioceptive acuity, Chiyohara et al. (2023)^25^ found that such training was more effective for participants who could better retain past sensory information in memory. No significant relationship emerged between learning capacity and proprioceptive acuity.

Is any of this information useful to future space travelers? Lackner & DiZio (2000)^8^ reported the observations of Schmitt & Reid (1985)^26^ of astronauts waking in the dark, unable to feel the locations of their arms. They could see a luminous dial floating in mid-air but were unaware it was the watch on their arm. This observation suggests that in microgravity spindle rates can fall to sufficiently low levels, where they are no longer capable of generating position sensations. Furthermore, it reinforces the view that in our everyday activities, on occasions where we are no longer able to see our limb, it is the spindle signal which provides the positional information, not any memory mechanism relating to where the limb was last placed.

If we are correct and a weak spindle signal in microgravity is the result of a fall in torque at the elbow joint^27^, re-establishing normal torque levels at the joint should recover position sense acuity. Recently, Motanova et al. (2022)^28^ described an axial loading suit designed for astronauts. The suit incorporates a system of elastic elements distributed according to the demands of chosen muscle groups. It was proposed that this would help recover the lack of proprioceptive feedback in microgravity. Such a suit targets, specifically, the position senses mediated by muscle spindles, since the memory mechanism of repositioning remains impervious to effects of changes in gravity.

Finally, some limitations of the present study should be highlighted. A typical problem of parabolic flight is the necessarily small sample sizes, which obviously limit the generalizability of the findings and the power of statistical procedures. Furthermore, it is conceivable that the findings obtained during the short, successive episodes of altered gravity are not transferable to conditions of continuous weightlessness, such as those found in space travel. However, that seems unlikely, given observations such as those of Schmitt & Reid (1985)^26^.

## Data Availability

The datasets generated during and analysed during the current study are available from the corresponding author on reasonable request.
